# Chloroquine Sensitizes Nasopharyngeal Carcinoma Cells but Not Nasoepithelial Cells to Irradiation by Blocking Autophagy

**DOI:** 10.1371/journal.pone.0166766

**Published:** 2016-11-30

**Authors:** Anna Makowska, Michael Eble, Kirsten Prescher, Mareike Hoß, Udo Kontny

**Affiliations:** 1 Division of Pediatric Hematology, Oncology and Stem Cell Transplantation, Medical Faculty, RWTH Aachen University, Aachen, Germany; 2 Department of Radiation Oncology, Medical Faculty, RWTH Aachen University, Aachen, Germany; 3 Electron Microscopic Facility, Institute of Pathology, Medical Faculty, RWTH Aachen University, Aachen, Germany; Swedish Neuroscience Institute, UNITED STATES

## Abstract

**Background:**

Treatment of nasopharyngeal carcinoma requires the application of high dosages of radiation, leading to severe long-term complications in the majority of patients. Sensitizing tumor cells to radiation could be a means to increase the therapeutic window of radiation. Nasopharyngeal carcinoma cells display alterations in autophagy and blockade of autophagy has been shown to sensitize them against chemotherapy.

**Methods:**

We investigated the effect of chloroquine, a known inhibitor of autophagy, on sensitization against radiation-induced apoptosis in a panel of five nasopharyngeal carcinoma cell lines and a SV40-transformed nasoepithelial cell line. Autophagy was measured by immunoblot of autophagy-related proteins, immunofluorescence of autophagosomic microvesicles and electron microscopy. Autophagy was blocked by siRNA against autophagy-related proteins 3, 5, 6 and 7 (ATG3, ATG5, ATG6 and ATG7).

**Results:**

Chloroquine sensitized four out of five nasopharyngeal cancer cell lines towards radiation-induced apoptosis. The sensitizing effect was based on the blockade of autophagy as inhibition of ATG3, ATG5, ATG6 and ATG7 by specific siRNA could substitute for the effect of chloroquine. No sensitization was seen in nasoepithelial cells.

**Conclusion:**

Chloroquine sensitizes nasopharyngeal carcinoma cells but not nasoepithelial cells towards radiation-induced apoptosis by blocking autophagy. Further studies in a mouse-xenograft model are warranted to substantiate this effect *in vivo*.

## Introduction

Nasopharyngeal carcinoma (NPC) is a highly malignant tumor arising from the epithelial lining of the nasopharynx. Epstein-Barr virus (EBV) appears to be the primary etiological agent in the pathogenesis in the endemic regions of South East Asia and in younger patients throughout the world. Contributory are environmental agents such as nitrosamines and tobacco as well as genetic factors [1–3]. Radiotherapy is the primary therapeutic modality for patients with NPC [4–5]. Whereas in adults, radiation doses required are around 70 Gy, such doses have been lowered in children and adolescents to about 60 Gy with the additional application of chemotherapy [6–8]. However, such doses still lead to major late toxicities in about 70% of patients such as xerostomia, neck fibrosis, dental caries, trismus, hypopituitarism, stunted growth, hearing loss and secondary malignancies [9–10]. Since the frequency and intensity of side effects correlate with the dose of radiation [11], the development of radiation sensitizer to further decrease the dose of radiation could be a useful means in order to decrease side effects while preserving tumor control.

Recently, autophagy has been shown to be deregulated in nasopharyngeal carcinoma cells and blockade of autophagy sensitized cells to apoptosis via cisplatin [12]. Autophagy is a self-degradative process in which cells eliminate intracellular aggregates and break down organelles as a source of fuel and metabolic precursors during conditions of stress such as nutrient deprivation, radio- or chemotherapy [13–14]. Autophagy is induced by formation of a unique flat membrane around damaged organelles or misfolded proteins, followed by establishment of a double-membraned autophagosome which then fuses with a lysosome to become the autophagolysosome. Its content is degraded by a series of lysosomal/vacuolar acid hydrolases. The resulting small molecules, particularly amino acids, are transported back to the cytosol for protein synthesis and maintenance of cellular functions. Autophagosome formation is dynamically regulated by a minimum of 16 autophagy-related proteins (ATG) [15–16].

Chloroquine has been used for decades as an antimalarial medication and more recently for the treatment of autoimmune disorders [17]. Chloroquine and its diastereoisomer hydroxychloroquine have been shown to be lysosomotropic agents that inhibit the activity of lysosomal enzymes by changing the pH, thereby inhibiting autophagy [18]. In preclinical models they have been found to sensitize tumor cells against DNA-damaging agents [19–20]. Recently, a phase I/II study in patients with glioblastoma multiforme showed that hydroxychloroquine could inhibit autophagy in vivo [21].

Since autophagy has been shown to be deregulated in NPC cells and radiation, in addition, induces autophagy [11,22], we investigated whether blocking of autophagy by chloroquine could sensitize NPC cells to radiation.

## Materials and Methods

### Cell lines and culture

Five NPC cell lines and one nasopharyngeal epithelial cell line as a control were used in this study. CNE-2 [23] and HONE-1 [24] cell lines were kindly supplied by Prof. Pierre Busson (Gustave Roussy Institute, Paris, France). Cell line HNE-1 [24] was obtained from Prof. Qian Tao from the Chinese University of Hong Kong, China. Cell lines CNE-1 [25] and C666-1 [26] were provided by Prof. Fei-Fei Liu (University of Toronto, Canada). The SV40T-antigen immortalized nasopharyngeal epithelial cell line NP69 [27] was supplied by Prof. George Tsao (The Chinese University of Hong Kong, Hong Kong, China).

EBV-negative cell lines CNE-1, CNE-2, HNE-1 and HONE-1 were cultured in Dulbecco’s modified Eagle’s Medium (PAN Biotech, Dorset, UK). The EBV-positive cell line C666-1 was maintained in RPMI1640 medium (Gibco, Paisley, UK). Both media were supplemented with 10% fetal bovine serum (Gibco, Paisley, UK), 100 U/ml penicillin and 100 mg/ml streptomycin (Gibco, NY, USA). Cells were cultured in a humidified incubator with 95% air and 5% CO_2_ at 37°C. The nasopharyngeal epithelium cell line NP69 was cultured in keratinocyte-serum free medium (Gibco, NY, USA).

Cells were exposed to photon-IR using a Synergy-Elekta irradiator (Stockholm, Sweden). Radiation was administrated as a single treatment or combined with chloroquine.

### Reagents

Chloroquine diphosphate was purchased from Sigma Aldrich (St. Louis, USA). The primary rabbit monoclonal antibodies against LC3-I, LC3-II, ATG3, ATG5, ATG6, ATG7 and β-actin were obtained from Cell Signaling (Danvers, MA, USA). The goat anti-mouse/rabbit IgG secondary antibodies were purchased from Santa Cruz Biotechnology (Heidelberg, Germany). The FITC-Active Caspase-3 Apoptosis Test was obtained from BD Pharmingen (San Diego, USA). For monitoring of autophagy in living cells by immunocytology and flow cytometry the Autophagy Detection Kit (Abcam, Cambridge, UK) was used. Hoechst 33258 was obtained from Sigma, Rotitest Vital from Roth (Karlsruhe, Germany). TUNEL assay was obtained from Roche (Mannheim, Germany). Lipofectamine 2000 was purchased from Invitrogen (Carlsbad, CA, USA) and siRNA oligonucleotides against ATG3, ATG5, ATG6, ATG7 and scrambled siRNA from Dharmacon, Lafayette, USA. Their sequences have been published before [28].

### Cell proliferation assay

The WST-8 reduction assay (Rotitest Vital) was used in preliminary experiments to choose the effective dose of radiation for subsequent experiments combining radiation with chloroquine. Cells were plated in 96-well plates at a density of 5.000 cells/well in 200 μl of growth medium. After overnight incubation, cells were irradiated at doses ranging from 0–8 Gy (S1 Fig). A radiation dose inhibiting about 50% of cell proliferation was chosen for further experiments. Secondly, the WST-8 reduction assay (Rotitest Vital) was used to determine the optimal concentration of chloroquine to inhibit autophagy with limited toxicity. The results indicated that chloroquine concentrations of 20 μM (S2 Fig) were non-toxic to cells and this dose was selected for further experiments. For experiments combining the effects of radiation and chloroquine, cells were incubated with chloroquine for 30 min and then irradiated. Following irradiation, cells were further incubated for 24 h, 48 h and 72 h and cell proliferation was analyzed adding 10 μl/well of WST-8 solution [2-(2-methoxy-4-nitrophenyl)-3-(4-nitrophenyl)-5-(2,4-disulfophenyl)-2H-tetrazolium, monosodium salt] for 4 h. Absorbance was then measured at 450 nm with an Elisa microplate reader.

### Cell cycle analysis

NPC cells were seeded at a density of 1.5 x 10^5^ into 6-well plates and grown in normal medium for 1 d. The following day, cells were treated as described before. After radiation and chloroquine treatment, cells were harvested after 24 h, 48 h, 72 h and 96 h. The samples were washed with phosphate-buffered saline (PBS) twice, centrifuged at 1.000 rpm for 5 min and resuspended in 0.5 ml fluorochrome solution containing: 0.1% sodium citrate, 0.1% Triton X-100, 50 mg/L propidium iodide in deionized/distilled water [29]. The cells were incubated 1 h in the dark at 4°C. Cell cycle distribution was analyzed by FACS (BD FACS Canto II, San Jose, CA) and at least 10.000 cells per reaction were counted. Results were analyzed by the FlowJo software, Oregon, USA. Three independent experiments were performed for each assay.

### Caspase-3 activity assay

For detection of active caspase-3, cells were pelleted, resuspended in 1 ml Cytofix/Cytoperm^™^ Fixation and Permeabilization Solution (BD Pharmingen, San Diego, USA) and incubated for 20 min on ice. The suspension was then centrifuged, and the pellet was washed twice with Perm/Wash^™^ Buffer (BD Pharmingen). Labeling was performed by adding to the cells 100 μl of washing buffer containing 20 μl of antibody (BD Pharmingen). The samples were incubated for 30 min at room temperature, washed in washing buffer and analyzed by flow cytometry. A negative control sample incubated with FITC-immunoglobulin G was run in parallel. Three independent experiments were performed for each assay.

### Chromatin staining with Hoechst 33258

Cells were prepared and treated as described. After radiation and chloroquine treatment, cells were harvested after further 72 h. Cells were then trypsinized and washed twice with PBS. The cell-permeant fluorescent marker Hoechst 33258 (1 μg/ml) was added to the cell pellet for 15 min at room temperature to assess the morphology of normal and apoptotic (i.e., condensed, fragmented) cells. Morphological changes of nuclei were observed under a fluorescence microscope (AMG, Mill Creek, USA), phase contrast images are shown to compare for cell density.

### TUNEL assay

The terminal deoxynucleotidyl transferase (TdT)-mediated dUTP nick end labeling (TUNEL) assay was carried out according to the manufacturer´s protocol (Roche, Mannheim, Germany). The fixed and permeabilized cells were incubated with FITC-12-dUTP, dATP and TDT to label apoptotic cells. Cells were then analyzed by flow cytometry.

### Autophagy detection

The Autophagy Detection Kit (Abcam) measures autophagic vacuoles in living cells using a dye that selectively labels autophagosomes [30]. For immunocytology, cells (5 x 10^4^) were grown on coverslips followed by radiation with and without chloroquine for 8 h. Cells then were washed and stained for autophagic vacuoles according to the manufacturer's instructions. Fluorescent autophagic vacuoles were immediately analyzed by fluorescence microscopy (AMG, Mill Creek, USA). For FACS analysis 1.5 x 10^5^ cells were prepared and treated as above, collected by trypsinization, centrifuged, resuspended in 250 μl of indicator free cell culture medium containing 5% FBS and 250 μl of the diluted Green stain solution and further processed according to the manufacturer's instructions. Samples were quantified by flow cytometry. Three independent experiments were performed for each assay.

### Immunoblot

Cells were treated as above, then collected and lysed with a buffer containing: 50 mM Tris-HCl (pH 7.4), 1% NP-40, 0.5% Na-deoxycholate, 0.1% SDS, 150 mM NaCl, 2 mM EDTA, 50 mM NaF and protease and phosphatase inhibitors. Equal amounts of protein were separated by SDS-Page and transferred on to a nitrocellulose membrane. After blocking, the membrane was incubated with primary rabbit monoclonal antibodies against LC3-I, LC3-II, ATG3, ATG5, ATG6, ATG7 and β-actin. After incubation with a goat anti-rabbit IgG secondary antibody, proteins were visualized. ATG3, ATG5, ATG6, ATG7, LC3-I and LC3-II protein expression levels were normalized with β-actin. The expression of LC3-I and LC3-II was quantified by ImageJ software (Wayne Rasband, NIH).

### Transmission electron microscopy

Cells were fixed in 3% glutaraldehyde (Agar scientific, Wetzlar, Germany) for at least 4 h, washed in 0.1 M Soerensen’s phosphate buffer (Merck, Darmstadt, Germany), and embedded into 2.5% agarose (Sigma, Steinheim, Germany). Agarose cubes were rinsed in 0.1 M Soerensen’s phosphate buffer. Post-fixation of cells was performed in 1% OsO_4_ in 17% sucrose buffer. After fixation, cells were washed in 17% sucrose buffer and deionized water and dehydrated by an ethanol series (30, 50, 70, 90 and 100%) for 10 min each and the last step thrice. The dehydrated specimens were incubated in propylene oxide (Serva, Heidelberg, Germany) for 30 min, in a mixture of Epon resin (Sigma) and propylene oxide (1:1) for 1 h and finally in pure Epon for 1 h. Samples were embedded in pure Epon. Epon polymerization was performed at 37°C for 12 h and then 80°C for 48 h. Ultrathin sections (70-100nm) were cut with a diamond knife (Leica, Wetzlar, Germany) by an ultramicrotome (Reichert Ultradcut S, Leica) and picked up on Cu/Rh grids (HR23 Maxtaform, Plano, Wetzlar, Germany). Negative staining by uranyl acetate and lead citrate (all EMS, Munich, Germany) was performed to enhance TEM contrast. The specimens were viewed using a Zeiss Leo-906 electron microscope (Oberkochen, Germany), operated at an acceleration voltage of 60kV.

### Transfection of siRNA

Transfection of siRNA was performed according to the protocol provided by the manufacturer (Invitrogen). Briefly, NPC cells were seeded at a density of 10^5^ cells / well in 24-well plates and allowed to adhere overnight. The following day, the cells reached about 80% confluence and were washed once with PBS and replaced with 500 μl of fresh growth medium without antibiotics. For each transfection, a suitable amount of siRNA was diluted in 100 μl Opti-MEM Medium. Lipofectamine (Invitrogen) was added directly to the diluted siRNA complex and incubated at room temperature for 5 minutes. The transfection mix was added into each well. After 16 h the transfection mix was removed and replaced with normal growth medium with antibiotics. The cells were treated as described before and incubated at 37°C with 5% CO_2_ for 72 h. Cell cycle analysis and detection of active caspase-3 were quantified by flow cytometry. Three independent experiments were performed for each assay.

### Statistical analysis

Experimental results were reported as a mean of at least three independent experiments conducted in quintuplicates for cell viability assays and triplicates for flow cytometric analyses. Data was expressed as the mean ± S.E. Significant differences between more than two groups were determined using ANOVA. Comparisons between two groups were performed using Student’s t-test. Differences were considered significant when the P value was less than 0.05.

## Results

### Chloroquine sensitizes nasopharyngeal carcinoma cells but not transformed nasopharyngeal epithelial cells against radiation

**Fig 1:**
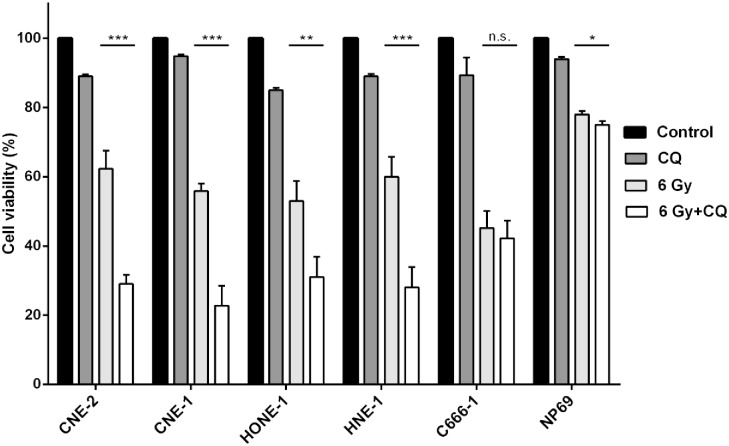
Chloroquine enhances the radiation-induced decrease of viability in NPC cells. Chloroquine further reduces the effect of radiation on cell viability in all NPC cell lines except of cell line C666-1. Only a weak effect is seen in the immortalized nasoepithelial cell line NP69. Cell proliferation was measured via WST-8 reduction Cells were plated in quintuplicates in 96-well plates. Data are presented as means ± S.E.M., each experiment was done three times (Student’s t-test; * = P<0.05; ** = P<0.01; *** = P<0.001).

Autophagy has been shown to protect cancer cells against the cytotoxic effect of chemo- and radiotherapy. In order to determine whether autophagy is an essential survival mechanism in nasopharyngeal carcinoma (NPC) cells following radiation, cells of five NPC cell lines were exposed to radiation in the absence or presence of the autophagy-inhibitor chloroquine and the percentage of living cells was determined after 24 h, 48 h and 72 h using the WST-8 reduction assay. In preliminary experiments a radiation dose of 6 Gy had been identified to significantly decrease cell survival in NPC cell lines by 40–70% after 72h (S1 Fig) and was chosen for the experiments. Similarly, different dosages of chloroquine were analyzed for their effect on viability in the NPC cell line panel. The dosage of 20 μM was found to decrease viability after 72 h by less than 10% and therefore used in the subsequent experiments (S1 Fig). When NPC cells were treated with 20 μM chloroquine half an hour prior to radiation, the percentage of living cells decreased up to additional 30% compared to cells treated by radiotherapy alone (Fig 1). Only in the NPC cell line C666-1 chloroquine did not further decrease the percentage of living cells after radiation. Compared to NPC cells, immortalized nasopharyngeal epithelial cells of cell line NP69 were less radiosensitive, with a reduction of viable cells by only 20%; adding of chloroquine before radiation did further decrease the percentage of living cells by only less than 5%. Since it has been previously shown that autophagy is up regulated in NPC cells [31–32], these results suggest that the sensitizing effect of chloroquine against radiation in NPC cells but not in nasoepithelial cells (NP69) could be based on blocking autophagy.

### Chloroquine enhances radiation-induced apoptosis in nasopharyngeal carcinoma cells

**Fig 2:**
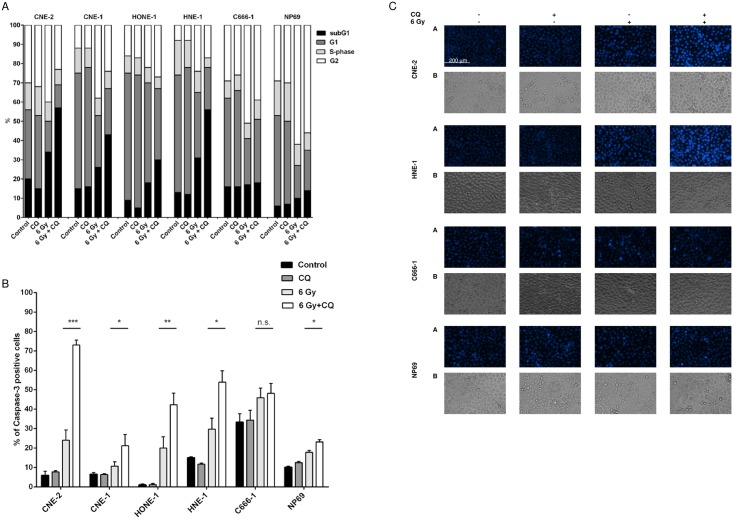
Chloroquine augments radiation-induced apoptosis in NPC cells. A) Cell cycle analysis. Radiation (6 Gy) induces a G2M arrest in all 5 NPC cell lines and the immortalized nasoepithelial cell line NP69. Radiation also induces apoptosis measured by an increase in subG1 in all NPC cell lines except of C666-1. Treatment with chloroquine (20 μM) significantly augments apoptosis in radiation-sensitive NPC cell lines compared to cells radiated only (CNE-2 and HNE-1: P<0.001; CNE-1: P<0.01; HONE-1: P<0.05; Student’s t-test). The data represent the means of three independent experiments and the corresponding standard error. (B) Combined treatment with chloroquine and radiation increases the number of cells with activated caspase-3 in radiation-sensitive NPC-cell lines. Quantitative data are reported as means ± S.E.M. (triplicate samples) (Student’s t- test; * = P<0.05; ** = P<0.01; *** = P<0.001). (C) Hoechst 33258 staining. Pretreatment with chloroquine before radiation increases the percentage of cells with morphological signs of apoptosis (condensed and fragmented nuclei) in radiation-sensitive NPC cell lines CNE-2 and HNE-1, but not in the radioresistant cell line C666-1 and the immortalized nasoepithelial cell line NP69. Morphologic changes were examined under a fluorescence microscope at 200x magnification, phase contrast images are shown for cell density comparison. Data of all experiments were shown at 72h after radiation.

In order to further analyze whether the significant lower percentage of living cells after treatment with radiation and chloroquine was due to increased cell death or proliferation arrest, cell cycle analysis was done. Radiation induced a G2/M arrest in all NPC cell lines and no difference was noted when cells were treated in addition with chloroquine. However, in cells treated with chloroquine and radiation, the percentage of cells with a subG1-DNA content significantly increased in all NPC cell lines except of C666-1, indicating a higher percentage of apoptosis (Fig 2a). Radiation induced apoptosis via activation of caspase-3, and again, except of cell line C666-1 cells pretreated with chloroquine had a higher percentage of activated caspase-3 than the ones, which had only been radiated (Fig 2b). In addition, apoptosis was measured by examining morphological changes in nuclei using Hoechst 33258 staining. As shown in Fig 2c, NPC cell lines CNE-2 and HNE-1 but not C666-1 showed the highest degree of morphological features of apoptosis, such as chromatin condensation and nuclear blebbing, when treated with radiation in the presence of chloroquine. Nasopharyngeal cells of cell line NP69 displayed similar to NPC-cell lines a G2/M-arrest after radiation, but only weak apoptosis which could only be slightly enhanced by chloroquine. Interestingly, whereas CNE-1 cells, irradiated and pretreated with chloroquine, showed a high percentage of apoptotic cells by subG1-analysis and Hoechst 33258 staining as well as by TUNEL-assay (S3 Fig), analysis of activated caspase-3 revealed only 21,19% positive cells, pointing out that execution of apoptosis in CNE-1 cells might only partly rely on caspase 3 [33–34].

### Chloroquine blocks autophagy induced by radiation in NPC cells

**Fig 3:**
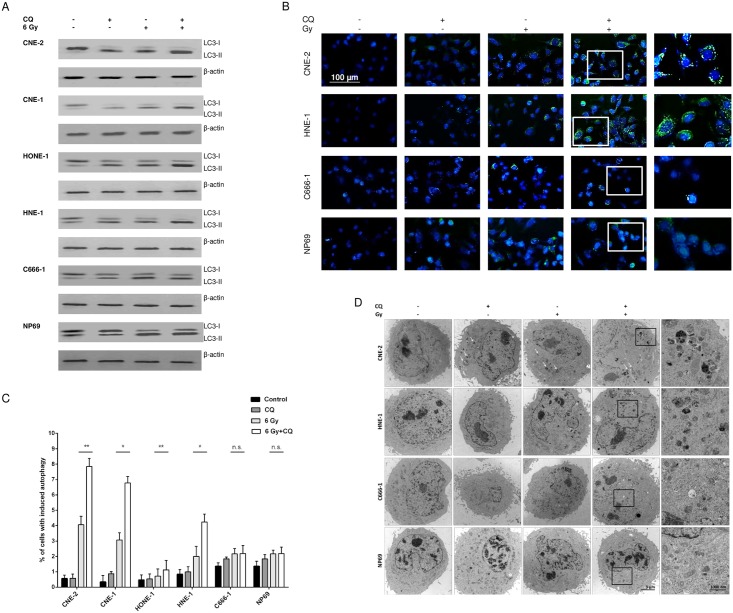
Chloroquine blocks radiation-induced autophagy in NPC cells. (A) Immunoblot for LC-I and LC-II. Preincubation with chloroquine before radiation increases expression of LC-II in radiation-sensitive NPC cells 8h post treatment (B) Immunofluorescence for autophagic vacuoles. Combined treatment of chloroquine and radiation increases the formation of autophagic vacuoles 8h post treatment in cell lines CNE-2 and HNE-1. Autophagic vacuoles were examined under a fluorescence microscope at 200x and 400x magnification. (C) Flow cytometric analysis of autophagic vacuoles. Combined treatment of chloroquine and radiation increases the number of cells with autophagic vacuoles 8h post treatment in radiation-sensitive NPC cell lines. (Student’s t- test; * = P<0.05; ** = P<0.01; *** = P<0.001) (D) Transmission electron microscopy. Photomicrographs show normal nuclear and mitochondrial morphologies in untreated cells and cells treated with chloroquine. Especially, in irradiated CNE-2 and HNE-1 cells the number of autophagosomes is significantly increased 8h following radiotherapy and further augmented by pretreatment with chloroquine.

To investigate whether the sensitization of NPC cells to radiation-induced apoptosis by chloroquine was associated with blocking autophagy, the following experiments were performed. First, the expression of LC3-I and LC3-II in NPC cells after treatment with radiation in the absence or presence of chloroquine was investigated by immunoblot. Of the four known LC3 isoforms, LC3-I and LC3-II are most widely used as autophagy markers. During autophagy, LC3-I is cleaved and conjugated to phosphatidylethanolamine. This modified form, termed LC3-II is involved in the closure of the autophagosome. After autophagosome-lysosome fusion, LC3-II is degraded and recycled to LC-I. The number of cellular autophagosomes and level of LC3-II expression is generally—but not always—indicative of autophagy induction. If autophagy is blocked with lysosomotropic reagents such as chloroquine, which inhibit acidification inside the lysosome or inhibit autophagosome-lysosome fusion, the degradation of LC3-II is blocked, resulting in the accumulation of LC3-II [35]. In our experiments, adding chloroquine to non-radiated cells increased LC3-II protein expression in all NPC cell lines compared to untreated controls indicating a basal level of autophagy (Fig 3a, S4a and S4b Fig). Similarly, radiation increased LC3-II expression in radiated NPC cells indicating induction of autophagy. LC3-II expression in radiated cells could be enhanced by chloroquine in all NPC cell lines except of C666-1. In normal nasopharyngeal epithelial cells radiation led to an increase in LC3-II expression whereas no effect of chloroquine could be observed on either non-radiated or radiated cells. The influence of radiation and chloroquine on autophagy in NPC cells was also analyzed by labeling autophagic vacuoles with a specific dye and analyzing them by immunocytology (Fig 3b) and flow cytometry (Fig 3c). Again, chloroquine increased the effect of radiation on the number of autophagic vacuoles in all NPC cell lines except in cell line C666-1, and in NP69-cells. Using transmission electron microscopy we were able to demonstrate intracellular autophagosomes in NPC cells 8 h following irradiation (Fig 3d). Treating cells with chloroquine prior to radiation increased the number of autophagosomes. However, in untreated cells or cells treated with chloroquine alone only few autophagosomes were observed. Taken together these results underline that chloroquine blocks autophagy induced by radiation in radiation-sensitive NPC cell lines.

### Inhibition of autophagy-related genes substitutes the enhancing effect of chloroquine on irradiation-induced apoptosis

**Fig 4:**
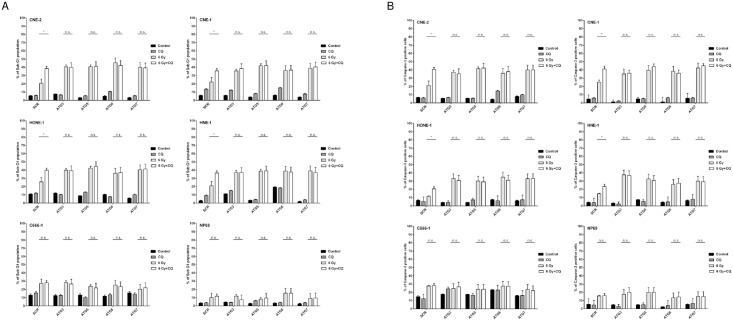
Knock-down of ATG3, ATG5, ATG6 or ATG7 by siRNA substitutes for the enhancing effect of chloroquine on radiation-induced apoptosis in NPC cells. Silencing of ATG3, ATG5, ATG6 or ATG7 by specific siRNA enhances radiation-induced apoptosis to a similar extent as chloroquine in radiation-sensitive NPC cell lines treated with scrambled siRNA as shown by an increase in subG1-DNA-content (A) or increase of active caspase-3 (B) both measured by flow cytometry. After transfection the cells were treated as described before. Quantitative data are reported as means ± S.E.M. (triplicate samples) (Student ‘s t- test; * = P<0.05).

Chloroquine can induce apoptosis via blocking autophagy or by autophagy-independent mechanisms such as inhibiting the degradation of PUMA [36–38]. In order to evaluate whether the observed increase in apoptosis of NPC cells by chloroquine when combined with radiation was due to the blockade of autophagy, expression of autophagy-related genes ATG3, ATG5, ATG6 and ATG7 was silenced by specific siRNAs and apoptosis was determined by analysis of subG1-DNA content and measurement of active caspase-3. The efficiency of siRNAs was indicated by western blot (S5 Fig). ATG3, ATG5, ATG6 and ATG7 are involved in the early steps of autophagosome formation [28, 39]. As shown in Fig 4a and 4b, silencing of ATG3, ATG5, ATG6 and ATG7 alone did not significantly increase apoptosis. However, when cells were irradiated a significant higher level of apoptosis was seen in cells treated with siRNA against ATG3, ATG5, ATG6 and ATG7 when compared to mock-treated controls. Silencing of ATG3, ATG5, ATG6 and ATG7 increased apoptosis to a similar degree than the addition of chloroquine to mock-treated controls. These results suggest that the sensitizing effect of chloroquine to radiation-induced apoptosis in NPC-cells is based on down-regulation of autophagy.

## Discussion

In this study, we demonstrate that chloroquine sensitizes nasopharyngeal carcinoma cells but not nasoepithelial cells to the cytotoxic effect of radiotherapy and that this effect is mediated by the blockade of autophagy.

Radiotherapy is the main therapeutic modality for the treatment of nasopharyngeal cancer. For local control doses up to 70 Gy are required [5, 9]. Such high doses, however, are associated with severe long term complications in the majority of patients [10–11]. Consequently, the use of a radio sensitizing agent could help to reduce the dose of radiation while preserving tumor control and decrease late effects. Radiotherapy has been shown in different tumor cell systems to induce autophagy [40–41]. Autophagy is an important mechanism for cells to balance sources of energy at critical times in development and response to stress factors. It can be cytoprotective, helping the cell to survive or it can be cytotoxic, leading to cell death [15–16]. Autophagy has been shown in various tumor cell systems, including hepatocellular carcinoma and glioblastoma to protect cells against the cytotoxic effect of radiation or chemotherapy [42–44]. In nasopharyngeal carcinoma, several studies have demonstrated the induction of autophagy by radiation mostly using one cell line [31–32, 45]. To obtain a more representative picture we have examined a panel of 5 different nasopharyngeal carcinoma cells and were able to detect induction of autophagy following radiation in all of them, even in the relative radioresistant cell line C666-1. In contrast, in SV40T-antigen transformed nasoepithelial cells used as a control, autophagy was only weakly induced after radiotherapy. This suggests that in nasopharyngeal carcinoma blocking of autophagy could increase the therapeutic window of radiation by selectively increasing the cytotoxicity of radiation to nasopharyngeal carcinoma cells.

Autophagy can be blocked by different agents such as 3-methyladenine (3-MA) or chloroquine [46–47]. The latter has been widely used as an anti-malarial and anti-rheumatic agent. It disrupts lysosome acidification, thereby blocking the maturation of autophagosomes [18]. Chloroquine has been shown *in vitro* and in animal models to block autophagy in various tumor cell systems and to sensitize cells against chemo- and radiotherapy [19–20]. A phase I-trial of hydroxychloroquine with dose-intense temozolomide in patients with advanced solid tumors and melanoma demonstrated that hydroxychloroquine was able to induce autophagic vacuoles in PBMCs at concentrations well tolerated by patients [48]. In addition, partial responses were observed in 14% and stable disease in 27% of patients with malignant melanoma. Recently, hydroxychloroquine significantly increased progression-free survival of patients with brain metastases from solid tumors in a phase II-study when added to 30 Gy of whole-brain irradiation (WBI) in comparison to patients only radiated (83.9% vs. 55.1%) [49]. In our cell line panel, chloroquine blocked autophagy following radiation in all five NPC cell lines and increased radiation-induced apoptosis in four of them. No increase in the percentage of apoptotic cells was observed in cell line C666-1 which itself was most resistant to the dose of radiation applied in the experiments. This suggests rather a defect in the apoptotic machinery in C666-1 cells than deregulation of the complex interplay between autophagy and apoptosis [50]. It also points out that chloroquine could sensitize the majority of NPC cells to radiation-induced apoptosis, but that there are still mechanism of resistance not to be overcome by this radiosensitizer.

Sensitization to radiation-induced apoptosis by chloroquine could be replaced by inhibiting autophagy through specific siRNA against ATG3, ATG5, ATG6 or ATG7, indicating that the sensitizing effect of chloroquine towards radiation-induced apoptosis was based on blocking of autophagy.

## Conclusion

Our results suggest that chloroquine could serve as a sensitizing agent to radiation in patients with nasopharyngeal cancer. Further studies in a mouse-xenograft model are warranted to confirm this effect *in vivo*.

## Supporting Information

S1 Fig Effect of radiation on cell viability. Cell viability decreases between 24h and 48h after radiation starting at 2 Gy in all 5 NPC cell lines and to a lesser extent in the immortalized nasoepithelial cell line NP69. Cells were plated in quintuplicates in 96-well plates and cell viability was determined by WST-8 reduction. Data are presented as means ± S.E.M, each experiment was done three times. The one way repeated measures ANOVA documented significant changes in the percentage of living cells starting 48h after radiation (ANOVA: * = P<0.05; ** = P<0.01; *** = P<0.001).
(PPTX)

S2 Fig Effect of chloroquine on cell viability. Cell viability decreases in all 5 NPC cell lines and in the immortalized nasoepithelial cell line NP69 with increasing dosages of chloroquine starting 24h after incubation. Cells were plated in quintuplicates in 96-well plates and cell viability was determined by WST-8 reduction. Data are presented as means ± S.E.M., each experiment was done three times (ANOVA: * = P<0.05; ** = P<0.01; *** = P<0.001).
(PPTX)

S3 Fig Chloroquine augments radiation-induced apoptosis in CNE-1 cells. Flow cytometric analysis using TUNEL assay of NPC-cell line CNE-1 and nasoepithelial cell line NP69. Combined treatment of chloroquine and radiation significantly increased apoptotic cells in CNE-1 but not NP69 cells. Data are presented as means ± S.E.M., each experiment was done three times (Student’s t- test;** = P<0.01).
(PPTX)

S4 Fig Immunoblot-based quantification of LC3-I and LC3-II expression in NPC cell lines and cell line NP69. Data show protein expression levels 8h following treatment. Expression levels of LC3-I (A) and LC3-II are normalized to ß-actin. Data are represented as a means ± S.E.M. from three different assays (Student’s t-test; * = P<0.05; ** = P<0.01; *** = P<0.001).
(PPTX)

S5 Fig Knockdown of different ATGs in NPC cells by small-interfering RNA (siRNA). Specific siRNAs (+) but not scrambled siRNA (-) suppress the expression of respective ATGs in NPC cell lines and cell line NP69. Proteins were harvested for immunoblots 72h after transfection.
(PPTX)
