# Supplementary figures and images for: Chloroquine Sensitizes Nasopharyngeal Carcinoma Cells but Not Nasoepithelial Cells to Irradiation by Blocking Autophagy
Source: PLoS One. 2016 Nov 30;11(11):e0166766. doi: 10.1371/journal.pone.0166766 (PMC5130215; doi:10.1371/journal.pone.0166766)

## Slide 1
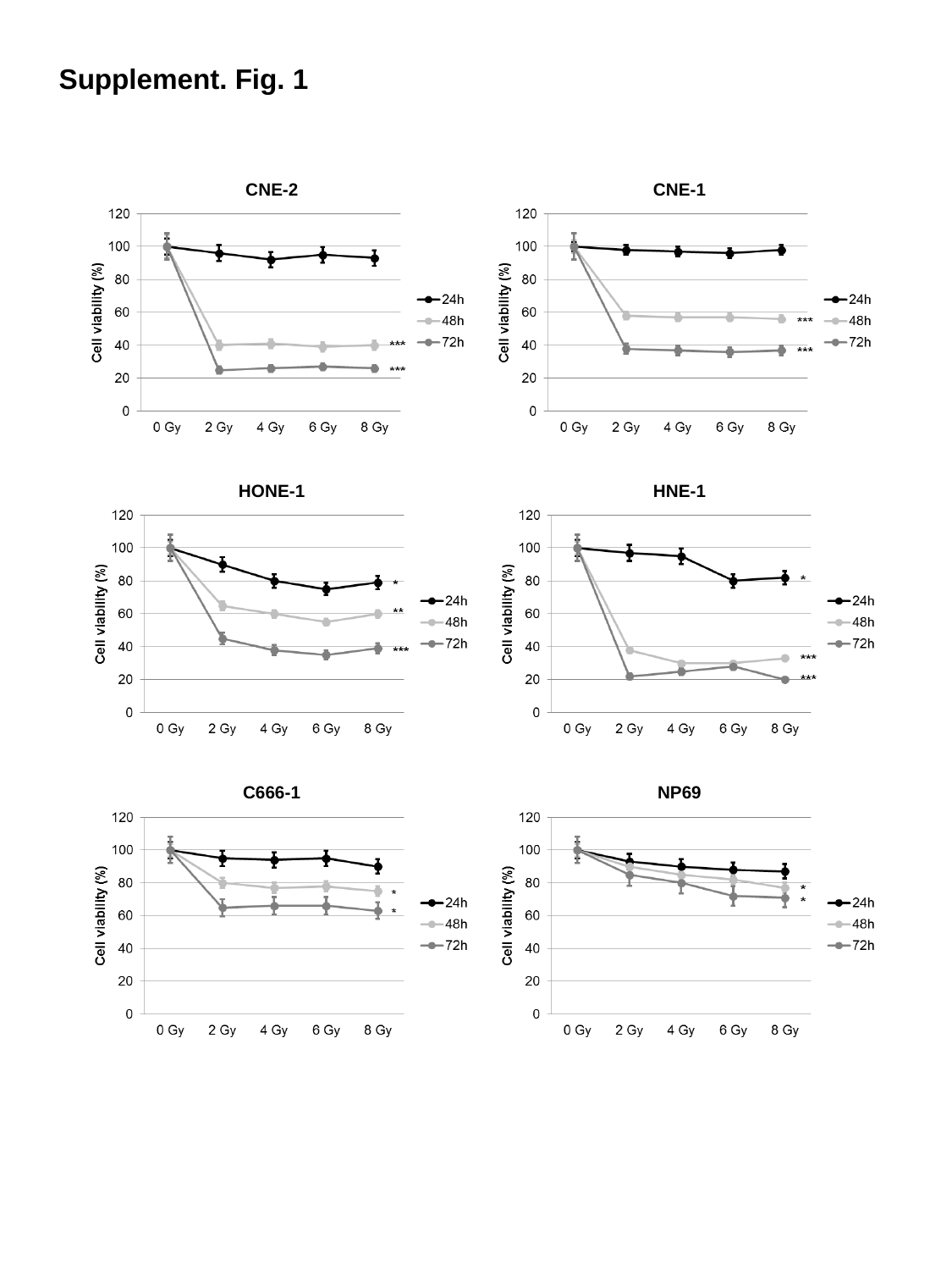

# Supplement. Fig. 1

Supplement: S1 Fig — Cell viability decreases between 24h and 48h after radiation starting at 2 Gy in all 5 NPC cell lines and to a lesser extent in the immortalized nasoepithelial cell line NP69. Cells were plated in quintuplicates in 96-well plates and cell viability was determined by WST-8 reduction. Data are presented as means ± S.E.M, each experiment was done three times. The one way repeated measures ANOVA documented significant changes in the percentage of living cells starting 48h after radiation (ANOVA: * = P<0.05; ** = P<0.01; *** = P<0.001). (PPTX) [file pone.0166766.s001.pptx]

## Slide 1
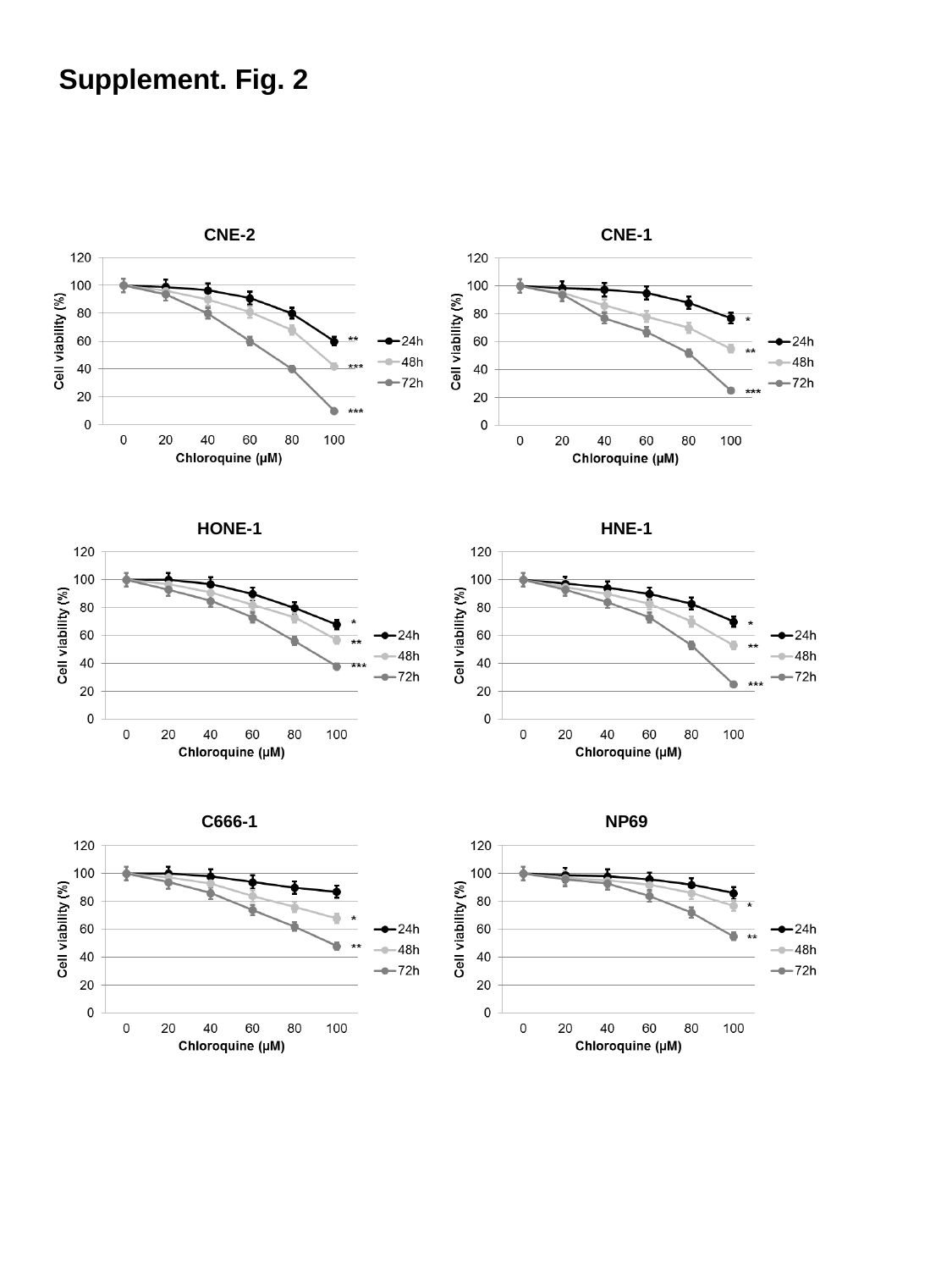

# Supplement. Fig. 2

Supplement: S2 Fig — Cell viability decreases in all 5 NPC cell lines and in the immortalized nasoepithelial cell line NP69 with increasing dosages of chloroquine starting 24h after incubation. Cells were plated in quintuplicates in 96-well plates and cell viability was determined by WST-8 reduction. Data are presented as means ± S.E.M., each experiment was done three times (ANOVA: * = P<0.05; ** = P<0.01; *** = P<0.001). (PPTX) [file pone.0166766.s002.pptx]

## Slide 1
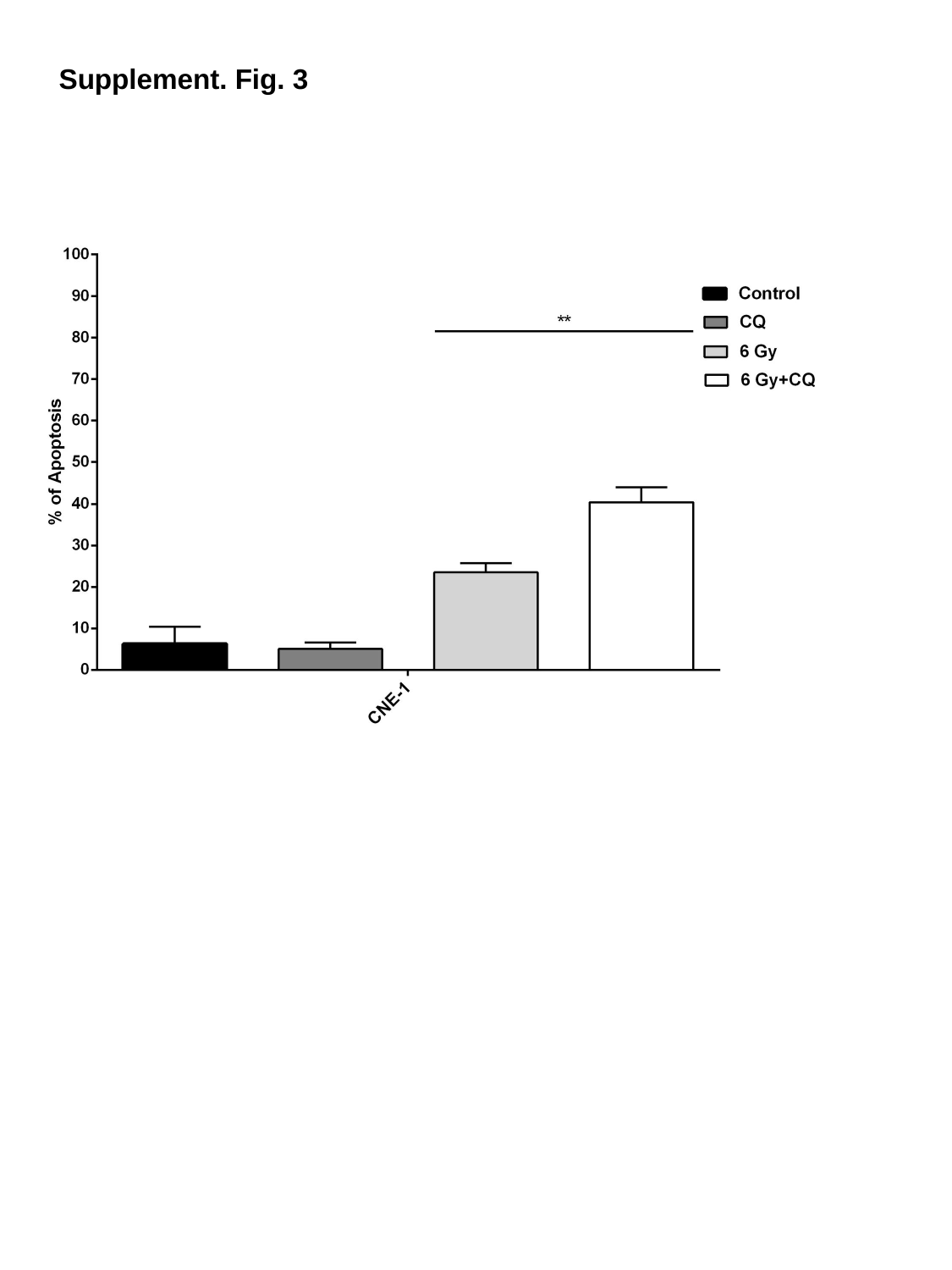

# Supplement. Fig. 3

Supplement: S3 Fig — Flow cytometric analysis using TUNEL assay of NPC-cell line CNE-1 and nasoepithelial cell line NP69. Combined treatment of chloroquine and radiation significantly increased apoptotic cells in CNE-1 but not NP69 cells. Data are presented as means ± S.E.M., each experiment was done three times (Student’s t- test;** = P<0.01). (PPTX) [file pone.0166766.s003.pptx]

## Slide 1
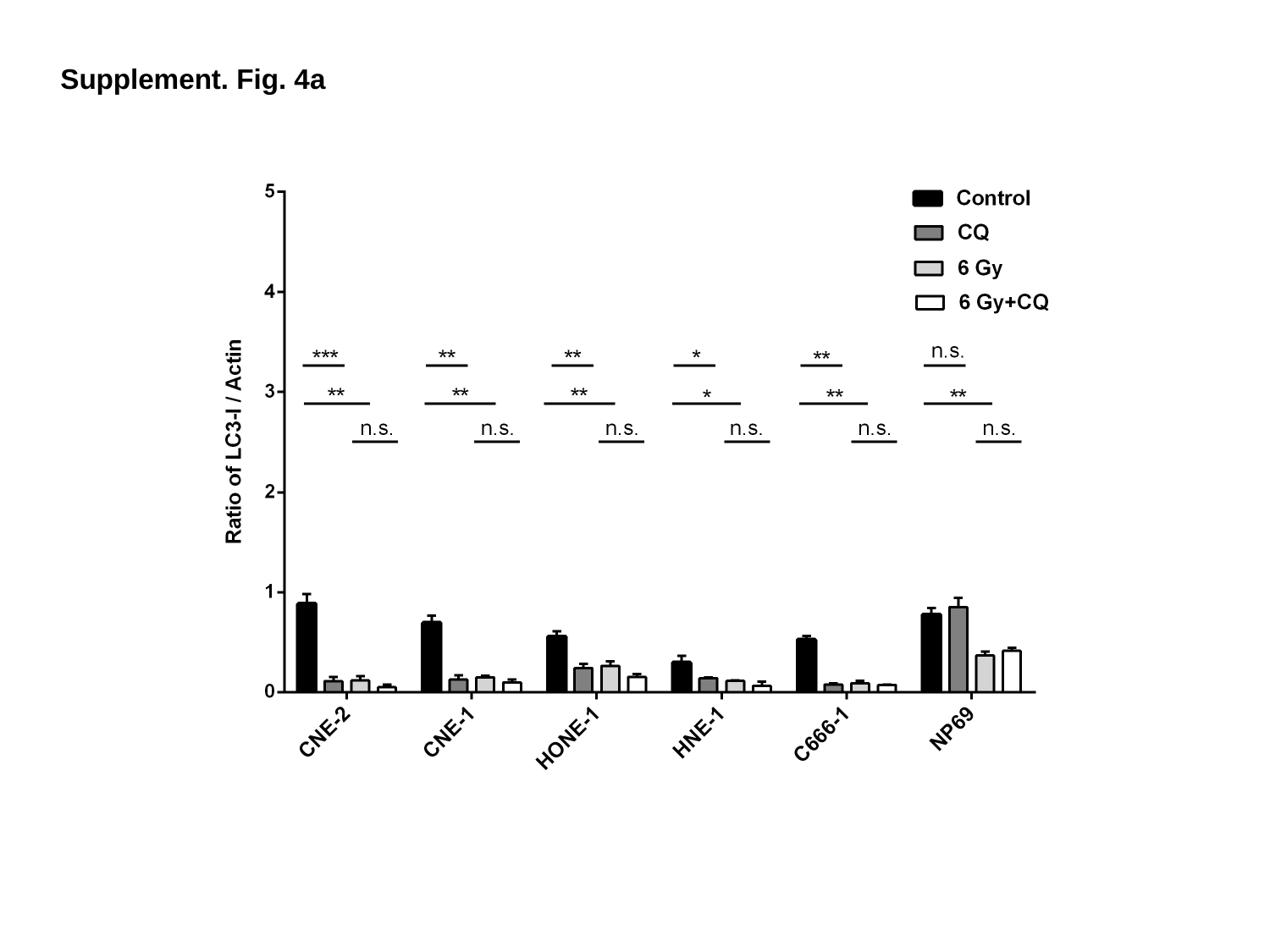

Supplement. Fig. 4a

## Slide 2
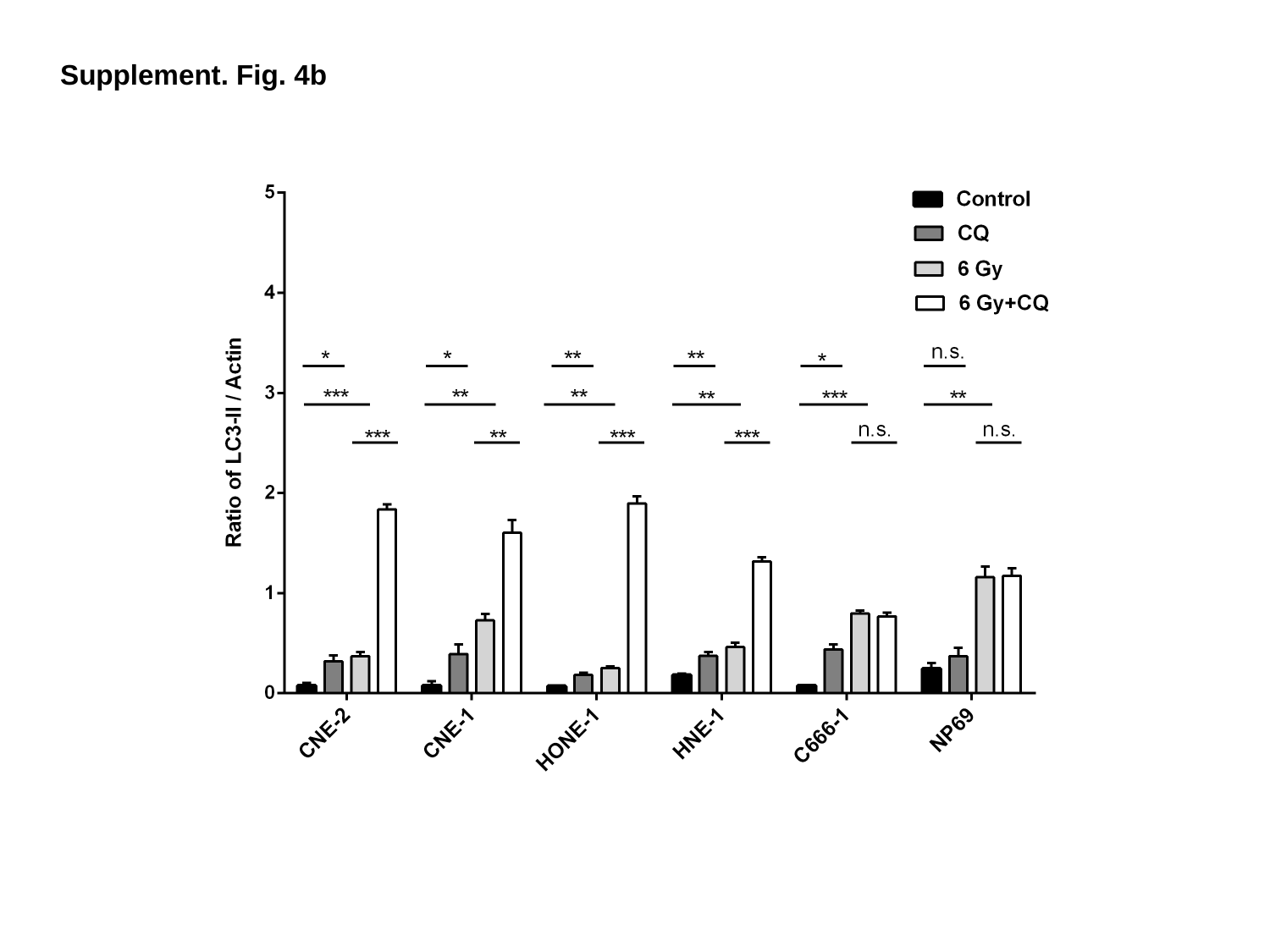

Supplement. Fig. 4b

Supplement: S4 Fig — Data show protein expression levels 8h following treatment. Expression levels of LC3-I (A) and LC3-II are normalized to ß-actin. Data are represented as a means ± S.E.M. from three different assays (Student’s t-test; * = P<0.05; ** = P<0.01; *** = P<0.001). (PPTX) [file pone.0166766.s004.pptx]

## Slide 1
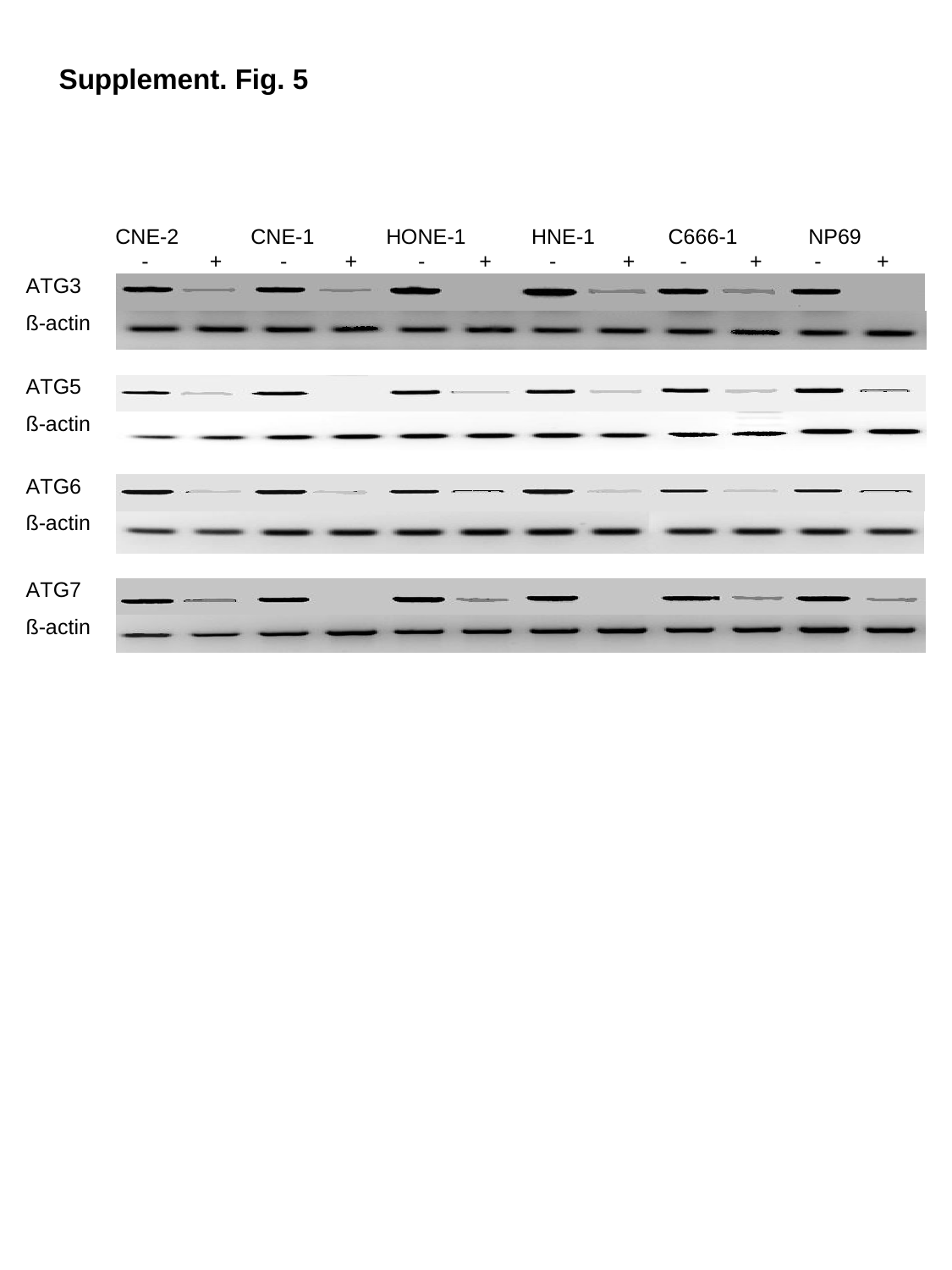

# Supplement. Fig. 5

Supplement: S5 Fig — Specific siRNAs (+) but not scrambled siRNA (-) suppress the expression of respective ATGs in NPC cell lines and cell line NP69. Proteins were harvested for immunoblots 72h after transfection. (PPTX) [file pone.0166766.s005.pptx]
